# Acute Auricular Perichondritis With an Effusion

**DOI:** 10.5811/cpcem.2019.9.43947

**Published:** 2019-10-21

**Authors:** Agnes Usoro, Michael R. Ehmann

**Affiliations:** Johns Hopkins, Department of Emergency Medicine, Baltimore, Maryland

## Abstract

A 62-year-old man presented to the emergency department with acute, atraumatic, swelling of his left ear. Incision and drainage revealed serous fluid without blood or purulence. He was diagnosed with acute perichondritis with an effusion and managed with oral antibiotics. Perichondritis must be recognized and treated promptly to avoid necrosis of the underlying avascular cartilage and auricular deformity.

## CASE PRESENTATION

**Image 1:**
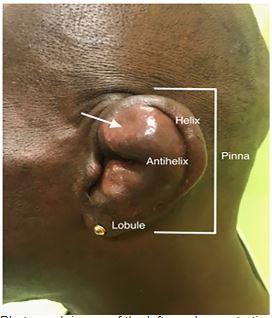
Photograph image of the left ear demonstrating swelling of the pinna (arrow) with mild overlying erythema.

**Image 2:**
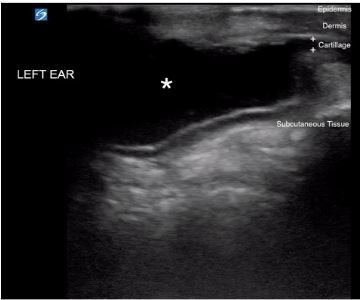
Ultrasound image (transverse view) of the left ear demonstrating an anechoic fluid collection (asterisk) within the cartilaginous layer (plus sign) of the pinna.

A 62-year-old man presented to the emergency department with one day of painful left ear swelling. He denied preceding trauma or recent instrumentation, but expressed concern that he might have been bitten by a spider while cleaning cobwebs in his basement two days prior. He denied systemic symptoms. The area of swelling was tender and fluctuant with mild overlying erythema (Image 1). Point-of-care ultrasound revealed an avascular anechoic fluid collection within the cartilaginous layer of the ear (Image 2).

Incision and drainage revealed serous fluid without blood or purulence. This fluid was sent for culture, and the incisional wound was closed with non-absorbable sutures. A xeroform bolster was then sutured through-and-through the contours of the antihelix with gauze buttressed behind the ear to prevent formation of an auricular hematoma. The patient was diagnosed with acute auricular perichondritis with an effusion and discharged with amoxicillin-clavulanate.

The patient’s culture grew methicillin-sensitive *Staphylococcus aureus*. He completed the course of antibiotics and had complete resolution of his symptoms and the fluid collection by his second otolaryngology follow-up appointment 14 days later.

## DISCUSSION

Acute auricular perichondritis is an infection of the pinna that involves the cartilage and subcutaneous tissue but spares the lobule. Most cases of perichondritis result from minor trauma, often after piercings or insect bites. *Pseudomonas aeruginosa* is the most common organism isolated but *Staphylococcus aureus* can also be causative, primarily after piercings.^1^–^3^ In the absence of infection, perichondritis – particularly recurrent perichondritis may herald underlying immunosuppression. 4,5

Perichondritis must be recognized and treated promptly to avoid necrosis of the underlying avascular cartilage and auricular deformity, better known as cauliflower ear.^1^,^3^ Treatment includes incision and drainage, auricular bolster placement and oral antibiotics with *Pseudomonas* coverage. All patients should follow up with otolaryngology for repeat wound evaluation and to ensure appropriate infection control.

**CPC-EM Capsule**

What do we already know about this clinical entity?

*Acute auricular perichondritis is an infection of the external ear that may result in auricular deformity and requires treatment with incision and drainage followed by oral antibiotics*.

What is the major impact of the image(s)?

*In addition to the typical external exam findings of auricular perichondritis, the ultrasound image is a unique example of how an auricular effusion appears sonographically*.

How might this improve emergency medicine practice?

*Sonographic evaluation of suspected auricular perichondritis may serve as a diagnostic adjunct when investigating if an auricular infection has an associated effusion requiring drainage*.

## References

[1] Prasad KC, Karthik S, Prasad SC (2005). A comprehensive study on lesions of the pinna. Am J Otolaryngol.

[2] Prasad HK, Sreedharan S, Prasad HS (2007). Perichondritis of the auricle and its management. J Laryngol Otol.

[3] Davidi E, Paz A, Duchman H (2011). Perichondritis of the auricle: analysis of 114 cases. Isr Med Assoc J.

[4] Levin RJ, Henick DH, Cohen AF (1995). Human immunodeficiency virus-associated non-Hodgkin’s lymphoma presenting as an auricular perichondritis. Otolaryngol Head Neck Surg.

[5] Caruso AM, Camacho M, Brietzke S (2014). Recurrent auricular perichondritis in a child as the initial manifestation of insulin-dependent diabetes mellitus: A case report. Ear Nose Throat J.

